# A Pilot Study in the Use of Activity Trackers for Assessing Response to Palliative Radiotherapy

**DOI:** 10.7759/cureus.1871

**Published:** 2017-11-22

**Authors:** Valérie Dorion, Louise Lambert, Alexandra Frazzi, Jean-François Cayer, Philip Wong

**Affiliations:** 1 Department of Radiation Oncology, Centre hospitalier de l'Université de Montréal (CHUM); 2 Unité De Recherche Clinique En Oncologie Et Hématologie, Centre hospitalier de l'Université de Montréal (CHUM)

**Keywords:** bone metastasis, radiation therapy, activity tracker, quality of life, prognosis, physical activity

## Abstract

Purpose

Radiation therapy (RT) has been a frequently used treatment for painful bone metastasis. The aim of this study was to determine the feasibility of using activity trackers (AT) to assess the patient prognosis and the effects of palliative RT.

Methods and materials

Twelve patients planned to receive palliative RT for axial metastases and were prospectively recruited to participate in this pilot clinical trial. The patients were eligible if there was no intent to change the analgesic medications prior to or within seven days of palliative RT. All the patients were lent a Misfit Flash^TM ^activity tracker (Misfit, Burlingame, California, United States of America) and were asked to wear it from the time of baseline assessment prior to RT until seven days after RT. The patients completed the European Organisation for Research and Treatment of Cancer quality of life (QOL) questionnaire (EORTC-QLQ C30) and the Short Form Brief Pain Inventory (SF-BPI) before the treatment and at days seven, 30 and 90 after completion of the RT. The patients' Karnofsky Performance Status (KPS) was assessed at each visit. The patients' overall survival at the end of the RT was recorded. Average daily steps before and after RT were compared using paired Wilcoxon signed-rank test. The patients' overall survival was estimated using the Kaplan-Meier curve and analyzed using the Log-Rank test.

Results

The median age of the patients was 62 years (range: 40-79 years). Of the 12 patients, there were five prostate, three breasts, three lungs, and one colon cancer-related patients. Six patients received 20 Gray (Gy) in five fractions and six received 8 Gy in one fraction. By day seven, post-RT, there was a 30% (p <0.02) reduction in the patients' daily activity level. There was no correlation between improvements in the QOL or with the level of pain and with the number of daily steps. While baseline KPS was not prognostic of the patient survival, the patients who on average took more than 7800 steps per day prior to RT lived significantly (p=0.034) longer than those who were less active.

Conclusions

The baseline activity level is associated with the patient prognosis. A significant decline in the physical activity was observed at one week after palliative RT. The use of activity trackers was to prognosticate and to monitor the patients' response to the palliative RT and should be evaluated further.

## Introduction

The palliative radiotherapy (RT) accounts for a significant proportion (between 25-40%) of the radiation oncologist’s practice [1]. The radiation therapy is an important adjunct in the interdisciplinary care of the palliative patients, as the RT can successfully reduce symptoms, most importantly pain, with fewer side effects [2-5]. While pain relief is often the primary goal of palliative RT, the ultimate objective is to improve quality of life (QOL), which may translate into improved well-being, function and mobility. Changes in pain and QOL are frequently measured subjectively using pain and QOL questionnaires [6]. Prior studies faced challenges, demonstrating the significant association between RT-related pain relief with the patient QOL as the patients often have multiple co-morbidities, progressive systemic diseases, and the treatment-induced toxicities [7]. In the secondary analysis of the Canadian Cancer Trials Group SC.23 phase 3 trial, Chow, et al. recently described an improvement in the functional interference and psychosocial aspects of the patients who reported a pain reduction, 10 days following palliative RT [8]. Objective measures of a patient’s physical functioning may complement currently available tools to better evaluate the effects of the palliative RT.

Selecting the intensity of the oncologic treatments, such as the fractionation schedule and complexity of technique, is frequently dependent on the perception of the patient’s prognosis [9]. However, the patient prognostication is often inaccurate and imprecise, and is mainly based on performance status scales such as Karnofsky Performance Status (KPS) and the Eastern Cooperative Oncology Group (ECOG) score, which represent crude estimates of a patient’s daily activity level as assessed by the physician at the time of the consultation [10-11]. Improving a clinician’s ability to estimate a patient’s lifespan could allow physicians to design palliative RT to an intensity and duration more suited to the patient.

Activity trackers (ATs) are wearable devices which monitor and track fitness-related metrics such as a person’s daily steps, heart rate, and even sleep patterns. These devices have surged in popularity, are becoming less costly ($20-100) and widely available. Many smartphones and watches have integrated health metric tracking. Pre- and post-intervention fitness metrics collected by ATs can be evaluated as an objective measure of a patient’s functional status. The activity trackers have been used in many oncological studies over the years, mainly to quantify the effects of chemotherapies on physical activity levels [12-13].

The aim of this pilot study was to determine the feasibility of using AT in assessing prognosis and the effects of palliative bone-related RT. The associations between the mean daily steps and physician-assessed KPS and the patient-reported outcomes (global QOL and pain) were explored.

## Materials and methods

The patients

The patients in this pilot study were recruited in a companion study to an ongoing prospective trial randomizing patients to receive palliative RT using either 3D-conventional radiation therapy (3D-CRT) or volumetric arc therapy (VMAT). This study was approved by the institutional ethics board. All eligible patients were adults with painful non-hematological cancer related axial metastases that necessitated palliative RT. At the time of registration of this study, the patients had a minimum KPS of 50 and were estimated to have a life expectancy of at least three months. The pain due to the metastases as measured using the Short Form Brief Pain Inventory (SF-BPI) was at least one out of 10. The patients also needed to complete the European Organisation for Research and Treatment of Cancer Quality of Life (QOL) questionnaire (EORTC-QLQ C30) to assess their QOL. The patients provided complete lists of their analgesics, which must not have been changed during the seven days prior to the palliative RT. During the seven days following the palliative RT, the patients were not expected to receive additional treatments (chemotherapy, RT or surgery). The patients KPS, analgesic intake, pain (SF-BPI) and the QOL (EORTC-QLQ C30) information were collected at baseline (prior to the RT), at seven, 30 and 90 days following the completion of the RT.

Activity tracker

The AT used for the study was the Misfit Flash^TM^ (Misfit, Burlingame, California, United States of America), which has a battery that lasts four to six months. The AT, which has 30 days of memory, continuously records the number of steps taken.

Study subjects, who had given informed consent, were lent an AT prior to receiving palliative RT. The patients were educated to wear the AT continuously prior to, during the course of RT and the week after palliative RT, except during water-related activities. At seven days post-RT, the ATs were returned to the investigative team and the data stored within the AT were extracted. As this was a pilot study to evaluate the patient acceptance and compliance with wearing ATs, we only asked the patient to wear them for seven days post-RT. Furthermore, considering our patient population, we were concerned that there would be a high attrition rate by one-month post-RT.

Quality of life

The EORTC-QLQ C30 is a validated questionnaire consisting of 30 questions to assess different functional and symptomatic aspects related to the patient QOL. In the current pilot study, only the last two questions from the questionnaire were analyzed to assess the global health status of the patient. Henceforth, all references to QOL will pertain to the global health status scores, which were calculated according to the EORTC scoring manual. Higher scores represent better global QOL. A ten point difference (one-100 scale) was determined a priority to be clinically important [14].

Pain

The perception of the pain was evaluated using the validated Short Form Brief Pain Inventory (SF-BPI) [15]. Through this questionnaire, the patients indicated their level and localization of pain prior to and at each subsequent visit following the palliative RT. The level of pain and confirmation of its location was analyzed to determine the patients’ response to the palliative RT. A variation of at least two points between two different tests was deemed as a clinically important improvement or worsening of pain if there has been no change in analgesic medication between the two tests [16].

Radiation therapy

All patients were CT-simulated. The palliative RT consisted of either 8 Gy in one fraction or 20 Gy in five fractions targeted to a painful metastatic site. The patients were treated to as the single region. The dose of RT was at the discretion of the treating physician. In the main study mentioned above, the patients were randomly assigned to be treated with 3D-CRT or VMAT.

Analysis

The number of daily steps was defined as the number of steps recorded by the AT on a fully tracked day. The average daily steps before and after the treatment were compared using paired Wilcoxon signed-rank test. All the data retrieved from the AT was normalized to the patients’ baseline values. The change in average daily steps from baseline to day seven was compared to the changes in the QOL, pain, and KPS from the same time periods. The patients' overall survival was estimated using the Kaplan-Meier curve and analyzed using the Log-Rank test. The statistical significance is defined by an alpha value of 0.05.

## Results

Twelve patients aged from 40 to 79 years old (median of 62) were recruited into this pilot study between November 2015 and August 2016 (Table 1).

**Table 1:** The characteristics of the 12 patients. The median (range) values are summarized in the last row. RT- Radiotherapy; f/u – Follow-up; M - Male; F - Female; Y - Yes; N - No.

Patient	Sex	Age	Cancer type	RT dose (Gy)	Pain flare	Number of days of recorded steps pre-RT	Number of days of recorded steps post-RT	Mean number of daily steps pre-RT	KPS Pre RT	Pain Pre-RT	Days from RT to death or last f/u
1	M	58	Prostate	8	Y	3	0	436	70	8	Alive: 544
2	M	61	Lung	8	Y	3	6	3819	80	6	Dead: 15
3	F	78	Breast	8	Y	0	10	n/a	70	5	Dead: 14
4	F	65	Lung	8	Y	1	7	3552	70	9	Dead: 269
5	F	40	Breast	20	N	3	7	8763	90	3,5	Alive: 581
6	M	67	Prostate	8	Y	5	7	7801	80	7	Alive: 555
7	F	64	Lung	20	N	0	5	n/a	70	8	Dead: 101
8	M	66	Colon	20	N	5	0	9460	90	6	Alive: 472
9	F	57	Breast	20	N	2	3	14743	80	8	Alive: 474
10	M	59	Prostate	8	N	0	1	n/a	70	10	Dead: 280
11	M	58	Prostate	20	N	5	8	7520	90	8	Dead: 189
12	M	79	Prostate	20	N	5	4	13030	90	8	Alive: 160
Median (range)		62 (40-79)				3 (0-5)	6 (0-10)	7801 (436-14743)	80 (70-90)	8 (3.5-10)	280 (14-81)

Among the 12 cases, there were five prostate, three breasts, three lungs, and one colon cancer patients. Six patients received 8 Gy delivered in one fraction and six patients were treated with 20 Gy delivered in five fractions. Five patients were treated with the spine (treated with one and six vertebrae). Six patients were treated to the pelvic region and one patient to the scapula. The pain flare was observed in five patients. The patients wore the AT for a median of three (zero-five) days before RT and for six (zero-10) days after the RT. Nine out of 12 patients wore the AT before the treatment and 10 out of 12 patients wore the AT after the treatment. One patient withdrew from the study, seven days after RT due to pneumonia (patient one). Two patients died 14 and 15 days after RT (patient two and patient three). The patients nine and 11 were patients who had previous irradiation on the main study and subsequently consented to this companion study at the time of re-irradiation and thus, SF-BPI, KPS, and the QOL were not collected from these patients.

**Figure 1:**
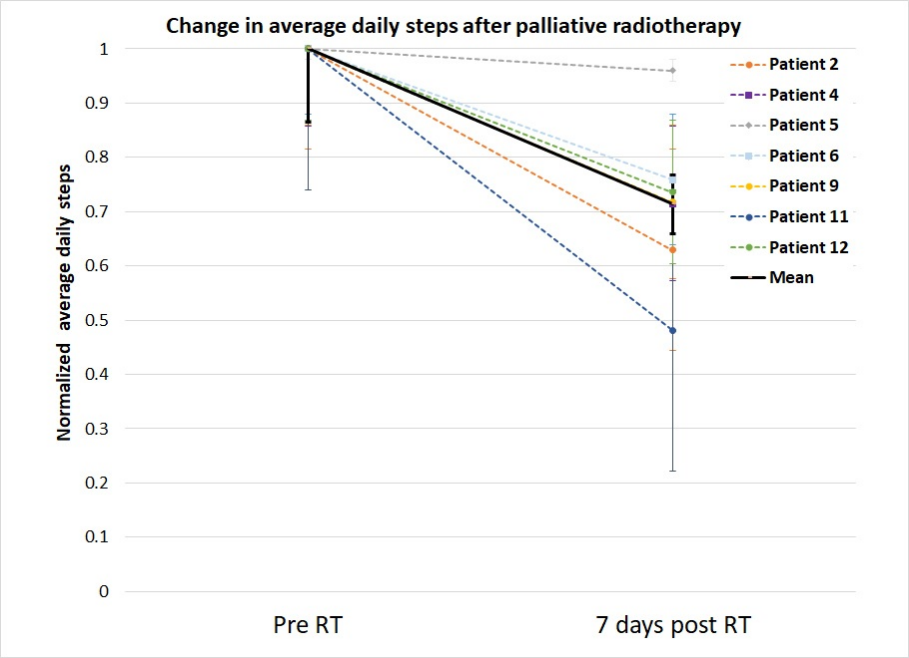
An average number of daily steps normalized to the patient activity level prior to the radiotherapy. The numbered steps prior to and after the radiotherapy (RT) taken by the patients were normalized to the average number of daily steps taken by the patients prior to the RT. Comparison of the daily activity level of the patient prior to (pre-RT) vs. after RT (seven days post-RT) were made using the Wilcoxon signed-rank test. Error bars represent standard error of the means.

Prior to the palliative RT, the median number of daily steps was 7800 (range: 436-14743 steps). Compared to pre-RT, the patients after palliative RT took 30% less daily steps, which was significant (Wilcoxon signed rank test p <0.02) (Figure 1).

**Figure 2:**
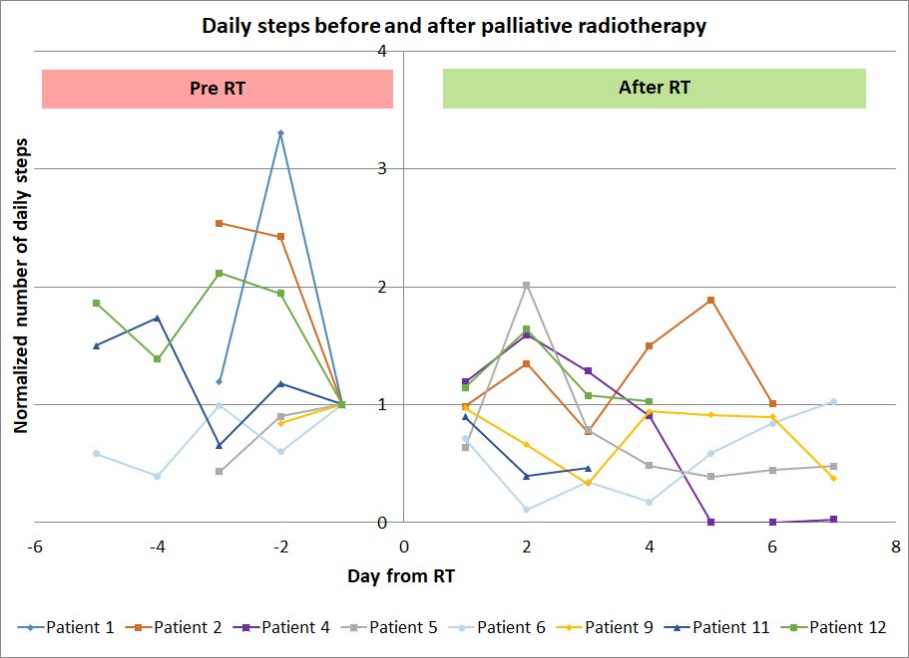
The normalized daily steps were taken by the patients before and after radiotherapy. The number steps prior to and after the radiotherapy (RT) taken by the patients were normalized to the average number of daily steps taken by the patients prior to the RT.

Figure 2 shows the daily variation of steps for each patient before and after the treatment.

**Figure 3:**
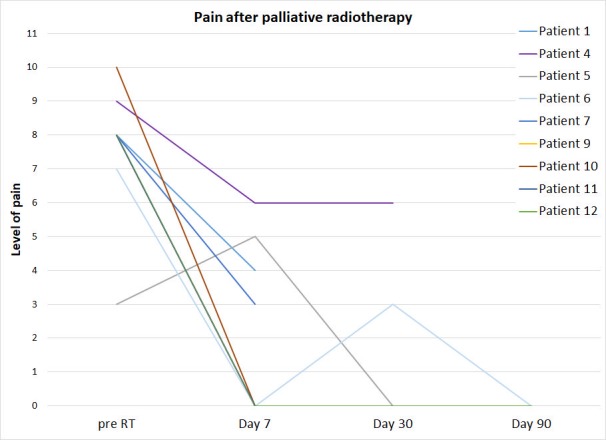
The patients' pain level prior to and after the palliative radiotherapy. Subjective pain level, based on the short form brief pain inventory, prior to the radiotherapy (pre-RT) and on day seven, 30 and 90 after the radiotherapy. The data points of the patients at day nine, 11 and 12 overlaps at pre-RT and day seven.

Eight out of the nine patients who responded to the SF-BPI had a clinically important (≥ 2 points) reduction in the pain without changes in opioid analgesics by day seven (Figure 3).

**Figure 4:**
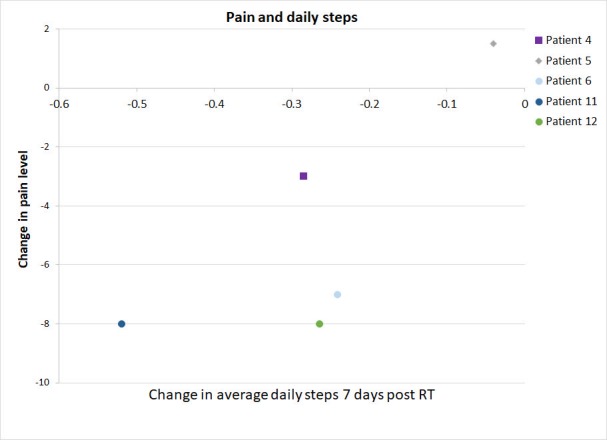
Change in the patients' pain level and activity level following the radiotherapy. Change in the patients, reported pain level (short form brief pain inventory) and average activity level following the radiotherapy (RT) are shown for the patients with both data available.

The EORTC-QLQ-C30 questionnaire was completed by six patients on day seven. There was no observable correlation between an improvement in the QOL and the number of steps (data not shown). There was no significant correlation observed between a reduction in the pain level and the change in the daily activity level (Figure 4).

**Figure 5:**
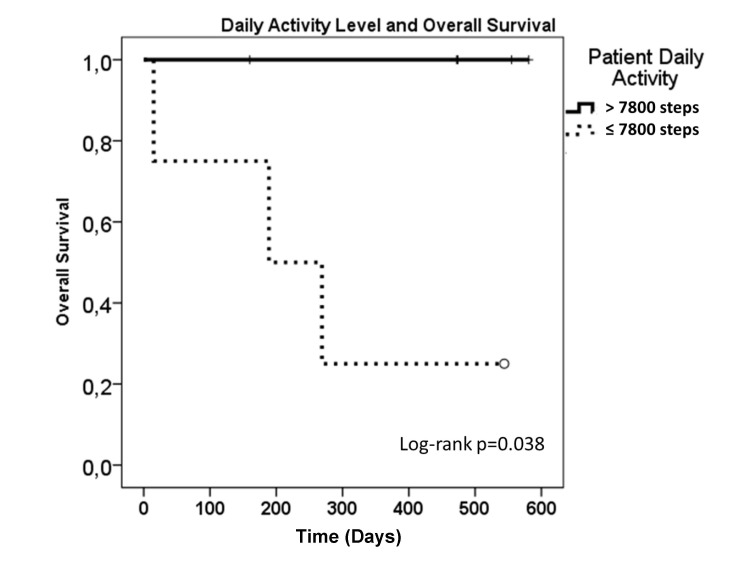
Overall survival of the patients as a function of baseline activity level. The average daily activity level (steps per day) was calculated for each patient (n=12). The patient groups were based on median patient activity level (7801 steps per day).

The general performance of the patients at baseline ranged from 70-90 KPS, or ECOG one. Although the patient KPS prior to palliative RT was not prognostic, the reduced activity level of the patients (≤ 7800 vs. > 7800 average steps per day) prior to RT was significantly (p=0.038) associated with overall survival (Figure 5).

## Discussion

This pilot study aimed to assess the feasibility of using AT to measure the impact of palliative RT on the patients' physical activity. There was a good acceptance from the patients in wearing ATs, as 11 out of 12 patients wore their ATs. However, the AT data was captured from 10 patients and due to the AT data loss, a common issue with the first generation ATs was reported [17]. We also could not differentiate when a patient was not wearing their ATs while sleeping. In accordance with previous reports [8], the clinically important improvement in the pain was observed in seven out of eight patients who completed the SF-BPI one week after receiving the palliative RT. Despite an improvement in the pain level, there was a significant decline in the physical activity of these patients (average daily steps) during the week after the treatment. There were too few QOL data to examine the correlation between changes in the QOL and physical activity.

Previous studies described the difficulty in estimating the prognosis of the patients receiving the palliative RT using conventional measures of the general performance status, such as KPS and ECOG [18-20]. In this study, we also could not differentiate the prognosis of the patients based on their ECOG or KPS. However, the patient baseline activity level was significantly (p=0.038) associated with the overall survival (Figure 5), suggesting that the AT data has the potential to better discriminate the patient prognosis than conventional measures of the performance status.

Although fatigue is a common side effect of the RT, we did not expect to see a 30% decline in the physical activity within one week of palliative RT, concurrently with an improvement of pain control. There are several possibilities to explain the decline in the physical activity after palliative RT. First, the treatment-related fatigue from the commute and RT may not be indolent. Although RT fatigue is usually low grade (grade one-two), the subtle increase in fatigue among the frail palliative patients could reduce their endurance to the physical activities without necessarily affecting their activities of daily living. Out of our 12 patients, five had pain flares (Table 1). Although all of the pain flares resolved by day seven, its occurrence could render the patients less active during and perhaps days after the incidence (Figure 2). Finally, another possibility could be related to the efficacious analgesic effect of the RT while maintaining the same opiate dose. A rapid decrease in the pain without reducing the dose of opioids could result in opioid overdose, sedation and subsequently a decrease in the activity level. This hypothesis seems to be supported by a non-significant inverse correlation between pain reduction and the patient activity, improved pain control seems to be associated with reduced steps taken by the patients (Figure 5). Due to the limited number of the patients who reported their QOL (n=4), the association of the QOL with other factors could not be properly derived.

The interpretation of the data from this study was limited because this was a pilot study of 12 patients. All patients had advanced cancers necessitating the palliative RT. One-third of the patients withdrew from the study before the 30-day follow-up. The precarious and declining conditions of the patients likely contributed to the low compliance rate in obtaining QOL and pain questionnaire data on follow-up. Finally, to ensure the short time frame between consultation and palliative RT, the number of days, the ATs were worn prior to RT was not controlled and was generally short. Therefore, the estimation of the physical activity level prior to RT might not be accurate.

Despite the above-mentioned limitations, our pilot study suggested a good acceptance from the patients in wearing ATs in spite of their advanced state of disease and discomfort. The compliance to wearing AT was better than the compliance in answering pain or the QOL questionnaires. The data loss seemed to be a less frequent issue in newer generations of ATs, which should be utilized in future studies. Although measuring the patients’ activity for a longer period prior to the treatment would better estimate the daily activity level, the palliative patients are often prioritized to receive the RT rapidly, such that some patients were recruited and treated on the same day (three out of 12 patients in this study). While postponing treatments to obtain more data prior to the RT would not be acceptable, future studies could lengthen the AT data collection period following RT beyond seven days to evaluate the time to the physical activity recovery following the RT.

## Conclusions

In conclusion, the palliative cancer patients were willing and compliant to wear the ATs prior to, during the palliative RT, and within the week after the treatment. Higher pre-treatment activity level was associated with longer overall survival. A significant decline in the physical activity was observed in the week after palliative RT was completed. Clinically important reduction in the pain level was observed one week after the palliative RT in the majority of the patients. The use of ATs to estimate the patient prognosis and to monitor the patient response to the palliative RT should be explored further using more reliable devices and longer follow-up periods.
